# High-intensity exercise improves multidimensional fatigue and health-related quality of life in rheumatoid arthritis: a randomized controlled study

**DOI:** 10.1186/s13075-025-03643-3

**Published:** 2025-09-18

**Authors:** Annelie Bilberg, Jan Bjersing, Mats Börjesson, Jenny Sivertsson, Kaisa Mannerkorpi

**Affiliations:** 1 Institution of Neuroscience and Physiology, Section of Health and Rehabilitation, Sahlgrenska Academy, University of Gothenburg, Gothenburg, Sweden; 2 Department of Occupational and Physiotherapy, Sahlgrenska University Hospital, Gothenburg, Sweden; 3 Institution of Medicine, Rheumatology and Inflammation Research, Sahlgrenska Academy, University of Gothenburg, Gothenburg, Sweden; 4 Department of Rheumatology, Sahlgrenska University Hospital, Gothenburg, Sweden; 5 Institution of Medicine Dept of Molecular and Clinical Medicine, Sahlgrenska Academy University of Gothenburg, Gothenburg, Sweden; 6 Center for Lifestyle Intervention, Dept of MGAÖ, Sahlgrenska University Hospital, Gothenburg, Sweden; 7 Department of Physiotherapy, NU Hospital Group, Uddevalla Hospital, Uddevalla, Sweden

**Keywords:** Exercise, Fatigue, Health-related quality of life, Mood, Pain, Rheumatoid arthritis, Sleep

## Abstract

**Background:**

Prolonged fatigue is prevalent in people with rheumatoid arthritis (RA), and physical activity is recommended as an adjunct treatment option to managing fatigue. However, the type and dose of physical activity is not established. The purpose of this randomized controlled multicenter study was to evaluate the effect of high-intensity interval training (HIIT) and strength exercise on fatigue, sleep, mood, pain and health-related quality of life in people with RA.

**Methods:**

In total, 87 participants diagnosed with RA, mean age 48 (SD 9.66) Years and 84% females, were randomly assigned to an intervention group (IG) (*n* = 43) performing supervised HIIT and strength exercise for 12 weeks, or a control group (*n* = 44) with counseling of the general physical activity recommendations. Self-administered measures were assessed at baseline, 3 months, 6 months and 12 months; the Multidimensional Fatigue Inventory (MFI 20), the Pittsburgh Sleep Quality Index (PSQI), the Hospital Anxiety and Depression Scale (HADS), pain (VAS), health-related quality of life (VAS-global). Disease activity was assessed with the DAS28-ESR. Statistical analyses were performed using Analysis of Covariance (ANCOVA), and mixed model repeated measure analysis.

**Results:**

At 3 months, a significant mean group difference in change was found on MFI-20 subscales General fatigue -4.0 (95%CI -5.57 to -2.39), Physical fatigue -4.9 (95%CI -6.43 to -3.36), Reduced activity -2.5 (95%CI -3.84 to -1.20), and Reduced motivation -1.8 (95%CI -2.97 to -0.62), favoring the IG. Additionally, a significant mean group difference in change was seen on VAS-global -12.8 (95%CI -21.3 to -4.3), in favor of the IG. At 6 months follow-up, the significant mean group difference on General fatigue -2.4 (95%CI -3.85 to -0.92), Physical fatigue -3.7 (95%CI -5.25 to -2.10), Reduced activity -2.0 (95%CI -3.53 to -0.39), Reduced motivation -1.6 (95%CI -2.93 to -0.16) and VAS-global -9.2 (95%CI -17.47 to -0.94) persisted. At 12 months follow-up a significant mean group difference was seen on Physical fatigue, depression mood, pain and VAS-global (*p*-value < 0.05), still in favor of the IG.

**Conclusions:**

The high-intensity exercise intervention had a beneficial effect on multidimensional fatigue, and health-related quality of life in people with RA. The effect on fatigue and health-related quality of life persisted during the 12 months-follow up period indicating long-term effects.

**Trial registration:**

The trial was registered prospectively on “FoU in Sweden” (Research and Development in Sweden), (registration number: 275642), and retrospectively on Trial Gov. (NCT 05768165).

**Supplementary Information:**

The online version contains supplementary material available at 10.1186/s13075-025-03643-3.

## Introduction

Rheumatoid arthritis (RA) is a systemic autoimmune disease affecting approximately 1% of the general population, and with a female predominance of 3:1 [1]. Persistent fatigue is a major problem among people with RA despite a more effective control of inflammation by improved pharmacological therapies [2]. The prevalence rate of fatigue in RA has been reported to be 40–80% [3, 4] which indicate a significant burden for the patient group. The phenomenon is multifaceted, varies over time and is described by many people with RA as an extreme feeling of tiredness [5]. Fatigue is suggested driven by physiological, psychological, behavioral, socio-cultural and temporal factors [6]. Moreover, fatigue is associated with disease activity, pain, and mood disorders [7], and many people with RA and persistent fatigue report sleep problem [8]. The consequences of fatigue may lead to deterioration in physical functioning [9] and health-related quality of life [10].

Exercise has the potential to improve disease activity [11] and physical function [12, 13] in RA. The general recommendations for physical activity, i.e. ≥ 150 min per week at moderate intensity or ≥ 75 min/week at vigorous intensity has been found to be safe for people with arthritis, thus recommended as part of the standard care of treatment [14]. Moreover, the European Alliance of Association for Rheumatology (EULAR) recommendations for people with inflammatory joint diseases and concomitant fatigue emphasizes tailored physical activity interventions as an adjunct option for managing fatigue [15]. However, the type and dose of the physical activity interventions are not established. There is some evidence that supervised aerobic exercise, at moderate-to-high intensity has positive short-term effects on fatigue in RA compared to no exercise [16, 17], although the quality of evidence is limited. Further, the effects of combined cardiorespiratory and strength exercise on fatigue show inconsistent results [18].

We have previously shown that a 12-week exercise intervention comprising supervised high-intensity interval training (HIIT) (≥ 90–95% HRmax) and strength exercise had beneficial effects on cardiovascular health and physical fitness without risk of deterioration in disease activity and pain [19]*.*

Although aerobic and strength exercise has demonstrated beneficial effects on physical fitness and health in people with RA, the impact of combined aerobic and strength exercise on fatigue are not fully elucidated. Hence, the effects of the high-intensity exercise intervention on fatigue should be explored as there is a clear need for effective strategies to alleviate fatigue in this patient group. The primary aim of this study was to evaluate the effect of supervised HIIT and strength exercise on multidimensional aspects of fatigue in people with RA. A secondary aim was to explore the effect of the exercise intervention on sleep quality, mood, pain, and health-related quality of life as these variables have previously been found to be associated with fatigue in RA. We also aimed to explore the associations between change in multidimensional fatigue and changes in sleep quality, mood, pain intensity, health-related quality of life and VO_2_max.

## Methods

### Study design

This is a secondary analysis of an assessor-blinded, two-armed multicentre randomized controlled trial [19], comparing the effects of 12 weeks supervised high-intensity exercise with counseling of the general recommendations of physical activity, in people with RA.

### Participants

The participants were recruited from hospitals in western Sweden, including the department of Rheumatology at Sahlgrenska University Hospital and the Rheumatology unit at the hospital of Uddevalla through the Swedish Quality Research Register. The recruitment, intervention and data collection were performed between August 2021 and March 2024. Patients between 20–60 years of age, fulfilling the 1987 American College of Rheumatology (ACR) or the 2010 European Alliance of Associations for Rheumatology (EULAR) criteria for RA [20], a disease duration > 1-year, stable treatment on antirheumatic drugs for the past 3 months, and low-to-moderate disease activity (DAS28) were eligible for participations. Exclusion criteria were symptomatic chronic coronary syndrome, pregnancy, other severe comorbidities and physical disabilities precluding participation in high-intensity exercise, and engagement in regular cardiorespiratory exercise on a high-intensity level (> 1 h/week for the past 6 months).

### Adverse effects and safety

An initial pre-screening was conducted using a protocol modified from the European Society of Cardiology’s (ESC) guidelines [21] to exclude individuals with symptomatic chronic coronary syndrome and severe comorbidities. This was followed by a medical evaluation and referral for a cardiopulmonary exercise test (CPET) for further screening of potential contraindications to high-intensity cardiorespiratory exercise.

### Exercise intervention

The exercise protocol has previously been described in more detail [19]. In brief, participants allocated to the intervention group (IG) performed supervised HIIT and strength exercise, two times per week for 12 weeks. The HIIT session on bicycles ergometric comprised high-intensity bouts (90–95% HRmax), interspersed with periods on lower intensity (70% HRmax). This was followed by strength exercise of large muscle groups 2–3 sets, 8–10 repetitions, at 70–80% of one repetition maximum (1RM). The exercise was individually tailored based on physical capacity and ability, and modified according to present symptoms and health. The exercise protocol followed the American College of Sports Medicine guidelines for cardiorespiratory and muscle strength exercise [22]. In addition, a third non-supervised cardiorespiratory session of the participant’s own choice was encouraged. Different motivational strategies were used during the exercise intervention including exercise guidance following the principles of self-efficacy and motivational support, identification of individual barriers for exercise, ongoing individual feedback by the physiotherapist, use of a personal exercise diary and heart rate monitoring. The physiotherapists that supervised the exercise were experienced clinicians in rheumatology. At the end of the intervention, participants were offered an individual session with a physiotherapist to set up a personalized plane for continuing health-enhancing physical activity/exercise in their own environment, following a person-centered approach [23].

### Control group

Participants in the control group (CG) received individual physiotherapy counselling on general physical activity guidelines, including encouragement to be active at a moderate intensity level ≥ 150 min per week. A home exercise program focusing on muscle strengthening exercises and balance was provided, along with verbal and written instructions.

In addition, standard outpatient care was given to all participants during the study period.

### Outcomes

Outcome measures were assessed at baseline and immediately after the intervention at three months, at six months and at 12 months follow-up including questionnaires using demographics, and medication use, along with a medical evaluation, performance-based tests and blood samples. All assessors i.e. physicians, physiotherapists, nurses and testing-personnel were blinded to group allocation at all study visits.

Primary outcome was multidimensional fatigue assessed with the Multidimensional Fatigue Inventory (MFI-20) scale [24], including five subscales; General fatigue, Physical fatigue, Mental fatigue, Reduced activity and Reduced motivation, ranging 4 to 20 for each subscale where a higher score indicates higher degree of fatigue. The MFI-20 has been frequently used in rheumatic diagnosis, RA included and is suggested to be reliable and valid for patients with rheumatic diseases [25].

Secondary outcomes were the following measures. Health-related quality of life and pain during the last week, caused by the rheumatic disease were scored with visual analogue scales (VAS), (0–100), where a higher score indicates worse health and pain. Sleep quality was assessed with the Pittsburgh Sleep Quality Index (PSQI) [26], including 19 items which generate seven components of sleep: sleep quality, sleep latency, sleep duration, habitual sleep efficiency, sleep disturbances, use of sleep medication, and daytime dysfunction. The seven components are summed into a global score (0–20) where a higher score indicate more severe sleep deprivations during the last month. A score > 5 is suggested to differentiate between normal and reduced sleep quality. Anxiety and depression were assessed with the Hospital Anxiety and Depression Scale (HADS) [27] which consists of 14 statements, ranging from 0 to 3, where a higher score refers to a higher degree of distress. The scores of the 14 items built two subscales: HADSa (0–21) for anxiety and HADSd (0–21) for depression [28]. Disease activity was assessed with the Disease activity score based on 28 joints (DAS28) [29], including clinical assessment of 28 joints (swollen, tender), patient´s rating of health (VAS), and erythrocytes sedimentation rate (ESR). Weight-adjusted VO_2_max (ml/min/kg) was obtained by a cardiopulmonary exercise test (CPET), during progressively increasing work on an ergometric bicycle with measured gas exchange [30]. Simultaneously, recording of 12 lead electrocardiogram data, heart rate, blood pressure and rating scores of symptoms (BORG 1–10) were assessed at baseline and at three months. The maximal obtained heart rate during the test was used as a reference during the cardiorespiratory exercises. Body mass index at baseline was calculated from measured body weight and body height (kg/m^2^). Activity limitations at baseline were assessed with the Health Assessment Questionnaire (HAQ) range 0–3 where a higher score indicates more activity limitations [31].

### Randomization

Randomization was performed after enrollment, with optimal allocation (minimization), using a computerized algorithm to balance for sex, age, VO_2_max, and study site. Participants were informed of their group allocation by the physiotherapist supervising the exercise.

### Statistical analyses

Descriptive statistics are presented as means and SD, frequency and percentages. The main statistical analysis between the two randomized groups was the Analysis of Covariance (ANCOVA) of change from baseline to three months for the effect variables adjusted for baseline value, age, sex and VO_2_max on the Full analysis set (FAS) population. We assessed the normality assumptions of the ANCOVA models by QQ-plots of the residuals. The first sensitivity analysis was the comparison between the change from baseline to three months for the effect variables analyzed with the Fisher´s non-parametric permutation test for continuous variables. The effect size was calculated as Cohen´s d coefficient, by the absolute difference in mean/pooled SD. Effect sizes 0.20 refers to a small change, 0.5 to a moderate change, and 0.8 or more to a large change. The secondary sensitivity analyses of primary efficacy variables were also performed on intention-to-treat (ITT) population using multiple imputation for missing values. Analyses of six- and 12-months follow-up were performed with a mixed model repeated measure model adjusted for baseline value, with unstructured covariance matrix and interaction term between group and visit. Comparisons within groups were performed with the Fisher’s non-parametric permutation test for paired observations for continuous variables and Sign test for dichotomous variables and ordered categorical variables. Correlations were calculated between change, baseline to 3 months in the intervention group for MFI-20 and change, baseline to 3 months for pre-selected variables of interest, using the Spearman’s correlation coefficient (r_s_). All tests were two-tailed and conducted at 0.05 significance level. All analyses were performed using SAS v9.2 (Cary, NC).

### Sample size

The sample size of 87 participants was based on the power calculation of change in VO_2_max, the primary outcome in the main study [19].

### Ethical

The study complied with the Declaration of Helsinki and was approved by the National Ethics Committee in Sweden (Dr nr; 2019–05255). Informed, written consent was obtained from all participants before baseline examinations. A patient research partner from the Swedish Rheumatism Association was involved in the planning and design of the study.

## Results

**Fig. 1:**
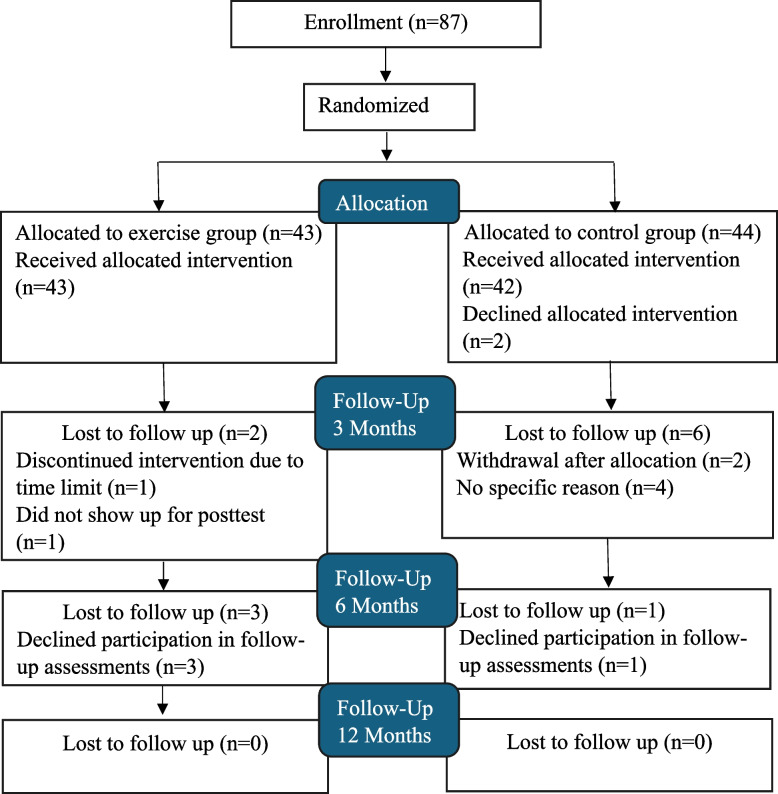
Consolidated Standards of Reporting Trials (CONSORT) diagram for the two groups in the randomised control trial

Demographics and clinical characteristics are presented by randomized groups in Table 1. In brief, out of 87 included participants, 84% were females. The mean age was 48 (SD 9.66) Years and mean disease duration at baseline was 6.7 (SD 4.98) years (range 1–25 years). According to DAS28 score, 23% of the participants had low-to-moderate disease activity and 77% were in remission. The participants reported substantial general and physical fatigue, along with reduced activity, mental fatigue and reduced motivation, at baseline (Table 2). The groups were considered similar in demographic and clinical characteristics at baseline. Flow chart of the patients is shown in Fig. 1.

**Table 1:** Baseline characteristics for the intervention group and the control group

	**Intervention (*n* = 43)**	**Controls (*n* = 44)**
Age, years	48.4 (10.1)	47.9 (9.3)
Sex		
Female	37 (86.0%)	36 (81.8%)
Male	6 (14.0%)	8 (18.2%)
Work status		
Full time, 80–100%	39 (90.7%)	40 (90.9%)
Part time, 1–79%	3 (7.0%)	2 (4.5%)
Not working	1 (2.3%)	2 (4.5%)
Education		
≤ 12 years,	14 (32.6%)	13 (29.5%)
High school, College, University	29 (67.4%)	31 (70.5%)
BMI-score	27.1 (5.3)	27.1 (5.3)
VO_2_mL/min/Kg	26.2 (5.3)	26.4 (6.5)
HAQ total, 0–3	0.4 (0.58)	0.4 (0.46)
Disease activity		
DAS28-ESR	2.0 (0.9)	2.0 (1.18)
Tender joints	0.6 (1.91)	1.1 (4.3)
Swollen joints	0.8 (1.59)	0.5 (1.0)
ESR, mm/hour	11.0 (11.2)	11.7 (10.1)
VAS-Pain, 0–100	20.0 (18.0)	20.0 (20.1)
VAS-Global, 0–100	21.0 (17.9)	18.8 (19.1)
Medication		
Conventional DMARD	34 (79.1%	32 (72.7%)
Biological DMARD	23 (53.5%)	21 (47.7%)
Targeted Synthetic DMARD	4 (9.3%)	3 (6.8%)
Corticosteroids (oral)	1 (2.3%)	2 (4.5%)
NSAID	17 (39.5%)	18 (40.9%)
Pain reliver, paracetamol	11 (25.6%)	12 (27.3%)

Categorical variables n (%) is presented and for continuous variables Means (SD)

BMI, Body Mass Index; VO_2_max, maximal oxygen consumption; HAQ, Health Assessment Questionnaire; DAS28, Disease Activity Score; ESR, Erythrocyte Sedimentation Rate; VAS, Visual Analogue Scale; NSAID, Non-steroidal Anti-inflammatory Drugs

**Table 2:** The effect of exercise on multidimensional fatigue from baseline (BL) to three months (3M) between the groups

	Intervention (*n* = 43)		Control (*n* = 44)		Between groups			
	BL Means (SD)	3 M Means (SD)	BL Means (SD)	3 M Means (SD)	Mean difference of change BL to 3 M, (95% CI), *p*-value^*^	Effect size	Adjusted mean difference of change BL to 3 M, (95% CI), *p*-value^§^	ITT analyses Mean difference of change BL to 3 M, (95% CI), *p*-value^‡^
MFI-20								
General Fatigue (4–20)	14.3 (3.6)	10.7 (4.0)	14.2 (3.8)	14.6 (3.9)	−4.0 (−5.81 to −2.22) < 0.0001	1.01	−4.0 (−5.57 to −2.39) < 0.0001	−4.0 (−5.57 to −2.35) < 0.0001
Physical Fatigue (4–20)	13.1 (4.6)	8.0 (3.15)	12.9 (4.1)	12.8 (4.5)	−4.9 (−6.83 to −3.05) < 0.0001	1.19	−4.9 (−6.43 to −3.36) < 0.0001	−4.9 (−6.43 to −3.32) < 0.0001
Reduced Activity (4–20)	11.1 (3.5)	8.4 (3.40)	11.2 (3.7)	11.1 (3.4)	−2.4 (−3.94 to −0.81) 0.0030	0.68	−2.5 (−3.84 to −1.20) 0.0003	−2.5 (−3.84 to −1.17) 0.0004
Reduced Motivation (4–20)	8.4 (2.91)	7.5 (2.97)	8.8 (3.52)	9.6 (3.52)	−1.7 (−2.96 to −0.40) 0.012	0.60	−1.8 (−2.97 to −0.62) 0.0032	−1.8 (−2.97 to −0.59) 0.0039
Mental Fatigue (4–20)	10.3 (3.8)	8.9 (3.90)	10.6 (3.5)	10.0 (3.07)	−1.4 (−2.95 to 0.13) 0.082	0.40	−1.2 (−2.58 to 0.14) 0.079	−1.2 (−2.58 to 0.18) 0.087

Effect size calculated as Cohen´s *d* coefficient mean difference/pooled SD

*MFI* the Multidimensional Fatigue Inventory scale

^§^Primary analyses performed with ANCOVA, adjusted for baseline value, age, sex and VO_2_max on FAS population

^*^Unadjusted sensitivity analyses with Fishers non-parametric permutation test on FAS population

^‡^Multiple stochastic imputation of 100 dataset adjusted for the minimization variables with ANCOVA, adjusted for baseline value, age, sex and VO_2_max

### Adherence and safety

A total of 33 participants in the IG followed ≥ 70% of the supervised exercise sessions. The drop-out rate during the intervention period was eight in total. In the IG, one participant withdrew after the first supervised session and one participant did not show up for post-test at three months. In the CG, two withdrew upon group allocation and four did not show up for post-test (Fig. 1). No clinical differences in sex, age and disease activity were observed between the participants that dropped out and those who completed the study. One participant in the IG experienced irregular heart rate during a supervised HIIT session and completed the intervention at moderately intensive level after consultation with a cardiologist and referral to UCG for evaluation of arrythmia. Four participants experienced temporary increases in musculoskeletal pain during strength exercise, which were managed through temporary exercise modifications. One participant experienced persistent musculoskeletal pain midway through the intervention and completed the intervention without the strength component. Two participants received a corticosteroid injection each, followed by temporary exercise modifications.

### The effect of the exercise intervention at three months

**Fig. 2:**
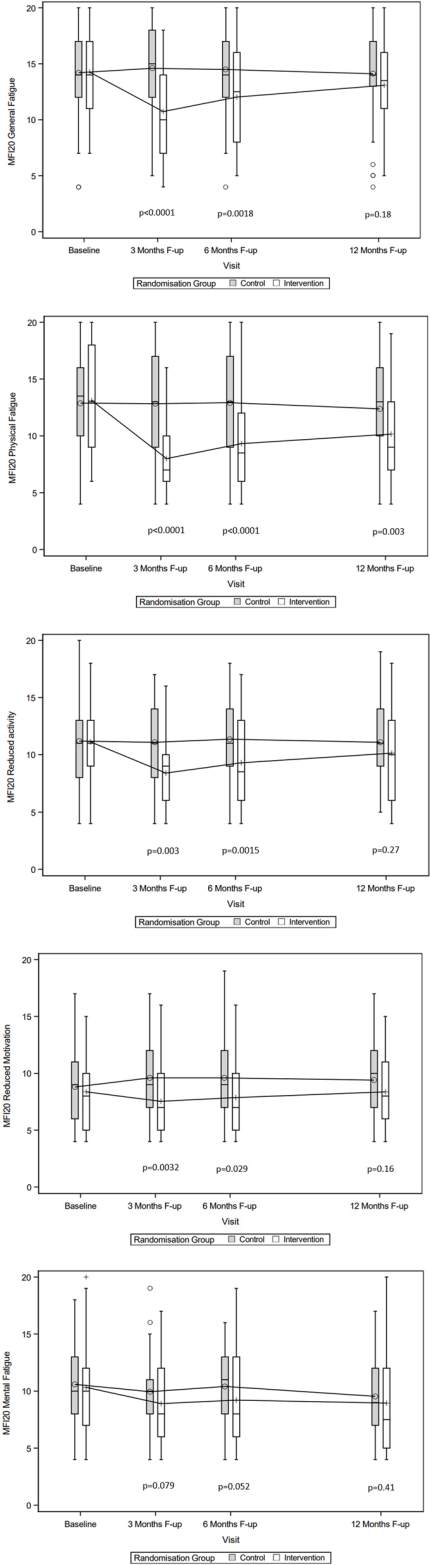
Distribution of the MFI-20 subscales in the groups at baseline, three, six and 12 months

A significant mean group difference of change was found on MFI-20 General fatigue −4.0 (95% CI −5.57 to −2.39), Physical fatigue −4.9 (95% CI −6.43 to −3.36), Reduced activity −2.5 (95% CI −3.84 to −1.20), and Reduced motivation −1.8 (95% CI −2.97 to −0.62), in favor of the IG. Moreover, the standardized effect size according to Cohen’s effect size index where large for General fatigue and Physical fatigue, and medium for Reduced activity and Reduced motivation. The sensitivity analysis on the ITT population revealed an equal mean difference of change for the separate MFI-20 subscales between the groups, see Table 2. The distribution over time of the MFI-20 sub scores in the IG and CG are illustrated in Fig. 2.

A significant mean group difference in change was found on VAS-global −12.8 (95% CI −21.3 to −4.3**)** in favor of the IG, Table 3. Within group analyses, baseline to three months for the IG and CG are presented in supplementary Table 1 and 2, se additional file.

**Table 3:** The effect of exercise on sleep, mood, health-related quality of life, pain and disease activity, baseline to three months

	Intervention group (*N*=43)		Control group (*N*=44)		Between group		
	BL Means (SD)	3 M Means (SD)	BL Means (SD)	3 M Means (SD)	Mean difference of change^b^ BL to 3M, (95% CI), *p*-value	Effect size	Adjusted mean difference of change^a^ BL to 3M, (95% CI), *p*-value
PSQI Global, 0–20	4.8 (2.99)	4.0 (2.68)	5.9 (3.63)	5.4 (2.77)	-0.7 (-1.82 to 0.40) 0.22	0.30	-1.0 (-2.0 to 0.01) 0.052
HADSa, 0–21	4.2 (3.61)	3.4 (3.19)	5.5 (3.89)	5.0 (3.47)	-0.7 (-2.0 to 0.53) 0.26	0.27	-1.0 (-2.18 to 0.10) 0.073
HADSd, 0–21	3.7 (2.56)	2.9 (2.55)	4.6 (3.69)	4.2 (3.59)	-0.8 (-2.16 to 0.47) 0.24	0.29	-1.1 (-2.26 to 0.12) 0.076
VAS-Global, 0–100	21.0 (17.9)	18.1 (16.7)	18.8 (19.1)	29.1 (26.5)	-14.0 (-23.0 to -5.1) 0.0016	0.70	-12.8 (-21.3 to -4.3) 0.0036
VAS-Pain, 0–100	20.0 (18.0)	18.9 (17.1)	19.9 (20.1)	21.4 (22.8)	-4.2 (-13.5 to 5.00) 0.37	0.20	-3.7 (-11.92 to 4.53) 0.37
DAS28-ESR	2.0 (0.90)	2.0 (0.82)	2.0 (1.18)	2.3 (1.33)	-0.2 (-0.60 to 0.10) 0.16	0.32	-0.3 (-0.61 to 0.01) 0.059
ESR	11.0 (11.2)	12.2 (10.4)	11.7 (10.1)	13.5 (11.4)	-0.6 (-3.23 to 1.90) 0.64	0.11	-0.8 (-3.12 to 1.56) 0.51

Effect size calculated as Cohen´s *d *coefficient, mean difference/pooled SD

*PSQI* the Pittsburgh Sleep Questionnaire Inventory global score, *HADSa/d* Hospital Anxiety and Depression subscale for anxiety and depression, *DAS28-ESR* Disease activity score based on 28 joints, *VAS* Visual Analogue Scale, *ESR* Erythrocyte Sedimentation Rate

^a^Main analyses of secondary variables performed with ANCOVA, adjusted for baseline value, age, sex and VO_2_max on FAS population

^b^Unadjusted analyses of secondary variables performed with Fishers non-parametric permutations test

### Medium-term and long-term effects of the exercise intervention

At six months, the mean group differences on multidimensional fatigue were still present, MFI-20 General fatigue −2.4 (95% CI-3.85 to −0.92), Physical fatigue −3.7 (95% CI −5.25 to −2.10), Reduced activity −2.0 (−3.53 to −0.39), and Reduced motivation −1.6 (95% CI −2.93 to −0.16), in favor of the IG. Moreover, the mean group difference on VAS-global −9.2 (95% CI −17.47 to −0.94) persisted, still in favor of the IG, Table 4.

**Table 4:** The effects of exercise on multidimensional fatigue, sleep, mood, health-related quality of life, pain, and disease activity, between baseline to six months and to 12-months follow-up

	Intervention (*n* = 38)	Controls (*n* = 37)	Between groups	Intervention (*n* = 38)	Controls (*n* = 37)	Between groups
	6 Months	6 Months	6 Months	12 Months	12 Months	12 Months
	Means (SD)	Means (SD)	Estimate mean diff (95% CI) *p*-value, effect size	Means (SD)	Means (SD)	Estimate mean diff (95% CI) *p*-value, effect size
MFI-20						
General fatigue, 4–20	12.0 (4.4)	14.5 (4.1)	−2.4 (−3.85 to −0.92) 0.0018 0.76	13.1 (3.9)	14.1 (4.2)	−1.0 (−2.34 to 0.46) 0.18 0.34
Physical fatigue, 4–20	9.32 (4.15)	12.9 (4.5)	−3.7 (−5.25 to −2.10) < 0.0001 0.99	10.2 (3.8)	12.4 (4.2)	−2.2 (−3.62 to -¸0.77) 0.0030 0.65
Reduced activity, 4–20	9.29 (3.78)	11.4 (4.2)	−2.0 (−3.53 to −0.39) 0.0151 0.50	10.1 (3.7)	11.1 (3.6)	−0.8 (−2.12 to 0.60) 0.27 0.25
Reduced motivation, 4–20	7.87 (3.44)	9.59 (3.58)	−1.6 (−2.93 to −0.16) 0.029 0.41	8.37 (3.02)	9.41 (3.48)	−0.8 (−1.99 to 0.33) 0.16 0.31
Mental fatigue, 4–20	9.21 (4.23)	10.4 (3.2)	−1.4 (−2.71 to 0.01) 0.052 0.46	8.95 (4.28)	9.54 (3.26)	−0.6 (−2.01 to 0.84) 0.41 0.20
PSQI Global, 0–20	4.28 (2.91)	5.42 (2.93)	−0.5 (−1.53 to 0.52) 0.33 0.03	4.50 (2.70)	5.67 (3.08)	−0.6 (−1.54 to 0.35) 0.21 0.13
HADSa, 0–21	4.13 (4.39)	5.86 (4.11)	−0.8 (−2.06 to 0.47) 0.80 0.22	3.16 (3.13)	5.19 (4.29)	−1.0 (−2.11 to 0.13) 0.082 0.31
HADSd, 0–21	3.29 (3.05)	4.64 (4.64)	−0.9 (−2.47 to 0.63) 0.24 0.21	3.16 (2.62)	4.78 (4.01)	−1.3 (−2.43 to −0.10) 0.034 0.41
VAS-Global, 0–100	18.7 (17.2)	26.3 (24.3)	−9.2 (−17.47 to −0.94) 0.030 0.51	16.3 (13.8)	28.7 (27.8)	−13.6 (−21.61 to −5.54) 0.0012 0.81
VAS-Pain, 0–100	17.8 (19.3)	25.3 (24.6)	−7.8 (−17.06 to 1.56) 0.10 0.39	15.0 (16.3)	27.0 (27.0)	−11.6 (−20.10 to—3.0) 0.0088 0.67
DAS28-ESR	2.2 (0.98)	2.5 (1.60)	−0.3 (−0.67 to 0.13) 0.18 0.29	2.2 (1.01)	2.4 (1.25)	−0.3 (−0.60 to 0.07) 0.12 0.33
ESR	11.2 (9.7)	12.8 (11.3)	−1.3 (−3.93 to 1.26) 0.31 0.16	13.3 (12.7)	12.2 (10.6)	0.9 (−3.24 to 5.10) 0.66 0.13

Mixed model repeated measure analysis with baseline value as covariate and visit six, and 12 months with unstructured covariance matrix and interaction term between group and visit

Effect size calculated as Cohen´s *d* coefficient mean difference/pooled SD

*MFI-20* Multidimensional Fatigue Inventory, *PSQI* Pittsburgh Sleep Quality Index, *HADSa/d* Hospital Anxiety and Depression subscale for anxiety and depression, *VAS* Visual Analogue Scale, *DAS28* Disease Activity Score based on 28 joints, *ESR* Erythrocytes Sedimentation Rate

At 12 months, the effect on Physical fatigue −2.2 (95% CI −3.62 to −0.77) and VAS-global −13.6 (95% CI −21.61 to −5.54) persisted, in favor of the IG. Moreover, a significant mean group difference of change was found on HADSd −1.3 (95% CI −2.43 to −0.10) and VAS-pain −11.6 (95% CI −20.10 to—3.0), in favor of the IG, Table 4.

### Associations between change in multidimensional fatigue and change in sleep, mood, health-related quality of life, pain and cardiorespiratory fitness in the intervention group at three months

The MFI-20 subscale General fatigue ∆, Physical fatigue∆ and Mental fatigue∆ had a moderate association with PSQI global∆ (r_s_ = 0.41 to 0.46) and a moderate to strong associations with HADSd∆ (r_s_ = 0.45 to 0.70). General Fatigue∆ and Physical fatigue∆ had a moderate association with VAS-pain∆ (r_s_ = 0.40 to 0.50) and a weak to moderate association with VAS-global∆ (r_s_ = 0.34 to 0.43). General and Physical fatigue∆ had a weak inverse association with VO_2_max∆ (r_s_ = −0.32 to −0.39017). All the above associations were significant (*p* < 0.05). The results of the correlation analysis for change in all primary MFI-20 are presented in Table 5.

**Table 5:** Associations between change in multidimensional fatigue, and change in sleep, mood, health-related quality of life, pain and VO_2_max three months in the intervention group

Variables	General fatigue∆ r_s_/*p* value (*n*=41)	Physical fatigue∆ r_s_/*p* value (*n*=41)	Reduced activity∆ r_s_/*p* value (*n*=41)	Reduced motivation∆ r_s_/*p* value (*n*=41)	Mental fatigue∆ r_s_/*p *value (*n*=41)
PSQI Global∆^*^	0.431 0.0059	0.415 0.0087	0.335 0.0370	0.477 0.0021	0.460 0.0032
HADSa∆	0.27 0.09	0.234 0.1399	0.389 0.0121	0.189 0.2367	0.243 0.1261
HADSd∆	0.70 <0.0001	0.452 0.0030	0.555 0.0002	0.577 <0.0001	0.550 0.0002
VAS-Global∆	0.34 0.0285	0.430 0.0050	0.414 0.0072	-0.006 0.9706	0.112 0.4841
VAS-Pain∆	0.40 0.0103	0.508 0.0007	0.475 0.0017	0.022 0.8916	0.168 0.2943
VO_2_ max∆	-0.39 0.0117	-0.322 0.0401	-0.098 0.5397	-0.079 0.6225	-0.294 0.0619

Spearman correlation coefficient (r_s_)

PSQI, Pittsburgh Sleep Quality Index; PSQI Global∆^*^ (n=39); HADSa/d, Hospital anxiety and depression subscale anxiety and depression; VO2max, Maximal oxygen consumption mL/min/kg; IG, Intervention Group

## Discussion

The randomized controlled trial demonstrates a beneficial effect on multidimensional fatigue after 12 weeks supervised HIIT and strength exercise in people with RA. The significant effect persisted at six- and 12-months follow-up which further support the effectiveness of the exercise intervention. In addition, a beneficial effect was found on health-related quality of life at all time points, favoring the IG. Disease activity remained stable in both groups throughout the 12-month period.

Fatigue is a complex, multidimensional phenomenon, and assessing the impact of interventions should encompass various aspects of fatigue [32]. The IG demonstrated significant improvement in the MFI-20 subscales General fatigue, Physical fatigue, Reduced activity and Reduced motivation compared to the CG at three months, supported by moderate to large, standardized effect sizes. These improvements persisted at six months, indicating a sustained medium-term effect. The impact of the exercise intervention on fatigue is to be compared with the small to moderate effects on fatigue reported of biological treatment in RA [2]*.*

The effect on physical fatigue in the IG was still present at 12 months follow-up when compared to the CG which has clinical implications. Physical fatigue is a strong predictor for physical inactivity in RA [33], and exercise has the potential to reduce fatigue [34]. The MFI-20 subscale Physical fatigue has been suggested to reflect physical ability to do things and physical condition [35], both of which are expected to improve with exercise. Accordingly, we observed a treatment effect in VO_2_max of 3.71 ml/min/kg accompanied by a 30% improvement in lower body strength, as previously reported [19]. Notably, contrary to previous findings reporting no associations between VO_2_max and fatigue [36, 37], a beneficial inverse association between change in VO_2_max and change in Physical fatigue and General fatigue was observed in the IG at three months. This suggest that the positive impact of the high-intensity exercise intervention on fatigue may partially be attributable to enhanced VO_2_max level. Improved VO_2_max and muscle strength has the potential to reduce systemic inflammation in arthritis [11, 38], which is shown to be bidirectional associated with fatigue and sleep in arthritis [18]. Although no change was seen in ESR in the present study, other inflammatory biomarkers might be associated with the improvement in fatigue [39, 40], warranting more research.

Sleep disorder is relatively common in RA and is found to be strongly associated with fatigue [8]. In the present study, a small although-non-significant change was found in sleep at three months, in favor of the IG. The mean difference in change on PSQI global is comparable to the results of a previous exercise study of people with RA reporting sleep disorders [41]. Still, more research is needed to evaluate the potential effect of exercise on sleep as conflicting results have been reported after aerobic exercise in arthritis [42, 43].

Variables previously found to be associated with fatigue such as pain and mood [9], and health-related quality of life [10] were also explored. A significant improvement in health-related quality of life was found at three months in favor of the IG, accompanied by a favorable trend toward enhanced mood. In adjunction, multiple significant associations between changes in MFI-20 subscales, and change in sleep, depression mood, pain and health-related quality of life were found within the IG. The positive effect on health-related quality of life persisted at 6 and 12 months, further accompanied by a significant improvement in depression mood and pain at 12 months, still in favor of the IG. Reduced fatigue and pain, along with improved ability to perform daily activities, have been identified as key facilitators of physical activity among people with arthritis [44]. It is likely that the intervention´s positive effects on physical fitness [19] and fatigue encouraged participants in the IG to maintain exercising, which in turn contributed to sustained improvements in multidimensional fatigue, reduced pain and enhanced mood, thereby reinforcing overall health.

Our findings add to the evidence of the positive effects of exercise in people with RA [18, 45]. The results are partly in agreement with previous meta-analysis reporting short-term effect of fatigue in RA after aerobic exercise [16, 17]. However, the effect on fatigue observed in the present study was larger than that reported in the meta-analysis. Furthermore, contrary to the results of the meta-analysis, the present study demonstrates small to large medium-term effects on fatigue at six months and a moderate long-term effect at 12 months, while no such effect was found in the meta-analysis. The differences in the results between the meta-analysis and the present study could partly be attributed to type and dose of the exercise interventions in the separate studies. However, a study conducted in axial spondyloarthritis, with similar type and dose of exercise as in the present study reported a small short-term effect on fatigue and no long-term effects at 12 months follow-up [43].

Significant within-group improvements were found on multidimensional fatigue in the IG, while no such effects were seen in the CG. Although no Minimal Clinical Important Difference (MCID) values of the sub scales of MFI-20 are established for RA, the interpretation of the mean differences of change reflect a clinically relevant effect as all the results except reduced motivation were substantial larger than suggested MCID for people with cancer [46], another patient group also affected of persistent and severe fatigue.

The disease activity remained stable during the study period in both groups indicating a low-to-moderate disease activity or clinically remission. The large majority of the study population (77%) were treated with conventional DMARD either as monotherapy or in combination. Additionally, 56% of the participants received biological treatment. Despite this, baseline ratings of general fatigue were substantial among the participants, which align with other studies of RA reporting elevated ratings of fatigue, also in well treated patients [47]. This suggests that the findings of this study could be generalized to other patient populations with RA.

### Clinical implications

Enhancing the quality of life for people with RA is emphasized. Given that fatigue is one of the most challenging aspects of RA treatment and a significant barrier to physical activity and exercise, the short-, medium-, and long-term effects on fatigue and health-related quality of life observed in the present study are important findings. The 12-week high-intensity exercise program of HIIT and strength exercise appear to be feasible and well tolerated, evidenced by high adherence to the protocol and few and limited adverse events. Moreover, the pragmatic approach employed to deliver the exercise in the study, utilizing existing exercise equipment at the physiotherapy units of the respective hospitals and clinically active physiotherapists supervising the exercise makes the implementation of the exercise protocol in clinical practice feasible.

## Strength and limitation

The major strength is the long-term randomized controlled design, the exercise protocol following the American College of Sports Medicine exercise guidelines for improved physical fitness [22], and the use of a multidimensional fatigue questionnaire. In addition, the well-defined study sample of out-patients from rheumatology units at two separate hospitals strengthens the generalizability of the results. However, the demanding exercise intervention may have led to the recruitment of highly motivated participants, potentially limiting generalizability to the broader RA population. A limitation is that no power calculation was performed on the primary outcome measures in the present study, potentially leading to insufficient power to detect true changes in fatigue. Nonetheless, there was a significant difference in change between the groups in the majority of the MFI-20 subscales at three and six months which indicate that the study sample can be considered sufficient.

## Conclusions

This is the first large scale randomized controlled study to our knowledge to demonstrate significant short-, medium- and long-term beneficial effects of a 12-week intervention of supervised HIIT and strength exercise on multidimensional fatigue, and health-related quality of life in people with RA. These findings support high-intensity exercise as a promising strategy for managing fatigue in this population.

## Supplementary Information

Additional file 1. Title: Supplementary Table 1. Changes in primary and secondary outcomes, baseline to three months in the intervention group. Description of data: Changes in primary and secondary outcomes baseline to three months within the intervention group.

Additional file 2. Title: Supplementary Table 2. Changes in primary and secondary outcomes, baseline to three months in the control group. Description of data: Changes in primary and secondary outcomes, baseline to three months within the control group.

## Data Availability

No datasets were generated or analysed during the current study.

## Abbreviations

ANCOVA: Analysis of Covariance
ACR: American College of Rheumatology
BMI: Body mass index
CPET: Cardiopulmonary Exercise Test
CRF: Cardiorespiratory fitness
cs/bDMARD: Conventional synthetic/biologic disease modifying anti-rheumatic drug
CV: Cardiovascular
DAS28-ESR: Disease Activity Score using 28 joint counts based on ESR; Erythrocyte Sedimentation Rate
ESC: European Society of Cardiology
EULAR: The European Alliance of Associations for Rheumatology
ESR: Erythrocyte sedimentation rate
FAS: Full analysis set population
HADS: The Hospital Anxiety and Depression Scale
HIIT: High-intensity interval training
ITT: Intention-To-Treat
MCID: Minimal Clinical Importance Difference
MFI-20: The Multidimensional Fatigue Inventory
NSAID: Non-steroidal anti-inflammatory drug
1RM: One Repetition Maximum
PSIQ: The Pittsburgh Sleep Quality Index
r_s_: Spearman’s rank correlation coefficient
RA: Rheumatoid arthritis
VAS: Visual analogue scale
VO_2_max: Maximal oxygen consumption
