# Supplementary material for: High-intensity exercise improves multidimensional fatigue and health-related quality of life in rheumatoid arthritis: a randomized controlled study
Source: Arthritis Res Ther. 2025 Sep 18;27:176. doi: 10.1186/s13075-025-03643-3 (PMC12447605; doi:10.1186/s13075-025-03643-3)
Supplement: Supplementary file 1 — Additional file 1. Title: Supplementary Table 1. Changes in primary and secondary outcomes, baseline to three months in the intervention group. Description of data: Changes in primary and secondary outcomes baseline to three months within the intervention group. [file 13075_2025_3643_MOESM1_ESM.docx]

| Table 1. Changes in primary and secondary outcomes, baseline to three months in the intervention group | | | | | |
| --- | --- | --- | --- | --- | --- |
|  | Baseline  (n=43)  Means (SD) | 3 months (n=41)  Means (SD) | Mean diff of change  (95%CI) | *p*-value | Effect size |
| MFI-20 |  |  |  |  |  |
| General  Fatigue | 14.3 (3.6) | 10.7 (4.0) | -3.5 (-4.90 to -2.07) | **<0.0001** | -1.00 |
| Physical  Fatigue | 13.1 (4.6) | 8.0 (3.15) | -5.1 (-6.59 to -3.56) | **<0.0001** | -1.11 |
| Reduced  activity | 11.1 (3.5) | 8.4 (3.40) | -2.6 (-3.81 to -1.36) | **0.0002** | -0.75 |
| Reduced  motivation | 8.4 (2.91) | 7.5 (2.97) | -0.8 (-1.78 to 0.12) | 0.097 | -0.28 |
| Mental  Fatigue | 10.3 (3.8) | 8.9 (3.90) | -1.2 (-2.45 to -0.04) | **0.042** | -0.33 |
| PSQI Global | 4.8 (2.99) | 4.0 (2.68) | -0.74 (-1.49 to 0.00) | 0.060 | -0.26 |
| HADSa | 4.2 (3.61) | 3.4 (3.19) | -0.66 (-1.36 to 0.04) | 0.075 | -0.18 |
| HADSd | 3.7 (2.56) | 2.9 (2.55) | -0.81 (-1.56 to -0.05) | **0.041** | -0.33 |
| VAS-global | 21.0 (17.9) | 18.1 (16.7) | -3.03 (-7.15 to 1.09) | 0.15 | -0.17 |
| VAS-pain | 20.0 (18.0) | 18.9 (17.1) | -1.73 (-6.87 to 3.42) | 0.51 | -0.10 |
| DAS28-ESR | 2.0 (0.90) | 2.0 (0.82) | -0.04 (-0.25 to 0.17) | 0.70 | -0.04 |
| ESR | 11.0 (11.2) | 12.2 (10.4) | 1.12 (-0.84 to 3.09) | 0.27 | 0.10 |
| Fisher’s non-parametric permutation test for paired observations for continuous variables and Sign test for ordered categorical variables.  Effect size mean difference/SD vid baseline  MFI-20, Multidimensional Fatigue Inventory; PSQI, Pittsburgh Sleep Quality Index; HADSa/d, Hospital Anxiety and Depression subscale for anxiety and depression; VAS, Visual Analogue Scale; DAS28, Disease activity score based on 28 joints; ESR, Erythrocytes sedimentation rate | | | | | |
