# Supplementary material for: High-intensity exercise improves multidimensional fatigue and health-related quality of life in rheumatoid arthritis: a randomized controlled study
Source: Arthritis Res Ther. 2025 Sep 18;27:176. doi: 10.1186/s13075-025-03643-3 (PMC12447605; doi:10.1186/s13075-025-03643-3)
Supplement: Supplementary file 2 — Additional file 2. Title: Supplementary Table 2. Changes in primary and secondary outcomes, baseline to three months in the control group. Description of data: Changes in primary and secondary outcomes, baseline to three months within the control group. [file 13075_2025_3643_MOESM2_ESM.docx]

| Table 2. Changes in primary and secondary outcomes, baseline to three months in the control group | | | | | |
| --- | --- | --- | --- | --- | --- |
|  | Baseline  (n=44) | 3 months (n=37) | Mean diff of change (95%CI) | *p*-value | Effect size |
| MFI-20 |  |  |  |  |  |
| General  Fatigue | 14.2 (3.8) | 14.6 (3.9) | 0.51 (-0.58 to 1.61) | 0.38 | 0.13 |
| Physical  Fatigue | 12.9 (4.1) | 12.8 (4.5) | -0.14 (-1.24 to 0.97) | 0.84 | -0.03 |
| Reduced  activity | 11.2 (3.7) | 11.1 (3.4) | -0.22 (-1.20 to 0.76) | 0.65 | -0.06 |
| Reduced  motivation | 8.80 (3.52) | 9.59 (3.52) | 0.86 (-0.01 to 1.74) | 0.055 | 0.24 |
| Mental  Fatigue | 10.6 (3.5) | 9.95 (3.07) | 0.14 (-0.84 to 1.11) | 0.83 | 0.04 |
| PSQI global | 5.88 (3.63) | 5.39 (2.77) | -0.03 (-0.88 to 0.83) | 1.00 | -0.01 |
| HADSa | 5.53 (3.89) | 5.05 (3.47) | 0.08 (-1.02 to 1.19) | 0.92 | 0.02 |
| HADSd | 4.65 (3.69) | 4.22 (3.59) | 0.03 (-1.13 to 1.18) | 0.99 | 0.01 |
| VAS-global | 18.8 (19.1) | 29.1 (26.5) | 11.0 (2.5 to 19.5) | **0.012** | 0.57 |
| VAS-pain | 19.9 (20.1) | 21.4 (22.8) | 2.50 (-5.75 to 10.75) | 0.56 | 0.13 |
| DAS28-ESR | 2.0 (1.18) | 2.3 (1.33) | 0.21 (-0.08 to 0.5) | 0.15 | 0.17 |
| ESR | 11.7 (10.1) | 13.5 (11.4) | 1.76 (0.06 to 3.47) | **0.044** | 0.17 |
| Fisher’s non-parametric permutation test for paired observations for continuous variables and Sign test for ordered categorical variables.  Effect size mean difference/SD vid baseline  MFI-20, Multidimensional Fatigue Inventory; PSQI, Pittsburgh Sleep Quality Index; HADSa,d, Hospital Anxiety and Depression subscale for anxiety and depression; VAS, Visual Analogue Scale; DAS28, Disease activity score based on 28 joints; ESR, Erythrocytes sedimentation rate | | | | | |
